# Microenvironmental cues enhance mesenchymal stem cell-mediated immunomodulation and regulatory T-cell expansion

**DOI:** 10.1371/journal.pone.0193178

**Published:** 2018-03-07

**Authors:** Rohini L. Kadle, Salma A. Abdou, Alvaro P. Villarreal-Ponce, Marc A. Soares, Darren L. Sultan, Joshua A. David, Jonathan Massie, William J. Rifkin, Piul Rabbani, Daniel J. Ceradini

**Affiliations:** NYU Langone Medical Center, Department of Plastic Surgery, New York, New York, United States of America; Universita degli Studi di Torino, ITALY

## Abstract

Mesenchymal stem cells (MSCs) are known to both have powerful immunosuppressive properties and promote allograft tolerance. Determining the environmental oxygen tension and inflammatory conditions under which MSCs are optimally primed for this immunosuppressive function is essential to their utilization in promoting graft tolerance. Of particular interest is the mechanisms governing the interaction between MSCs and regulatory T cells (Tregs), which is relatively unknown. We performed our experiments utilizing rat bone marrow derived MSCs. We observed that priming MSCs in hypoxia promotes maintenance of stem-like characteristics, with greater expression of typical MSC cell-surface markers, increased proliferation, and maintenance of differentiation potential. Addition of autologous MSCs to CD4+/allogeneic endothelial cell (EC) co-culture increases regulatory T cell (Treg) proliferation, which is further enhanced when MSCs are primed in hypoxia. Furthermore, MSC-mediated Treg expansion does not require direct cell-cell contact. The expression of indolamine 2,3-dioxygenase, a mediator of MSC immunomodulation, increases when MSCs are primed in hypoxia, and inhibition of IDO significantly decreases the expansion of Tregs. Priming with inflammatory cytokines IFNγ and TNFα increases also expression of markers associated with MSC immunomodulatory function, but decreases MSC proliferation. The expression of IDO also increases when MSCs are primed with inflammatory cytokines. However, there is no increase in Treg expansion when MSCs are primed with IFNγ, suggesting an alternate mechanism for inflammatory-stimulated MSC immunomodulation. Overall, these results suggest that MSCs primed in hypoxia or inflammatory conditions are optimally primed for immunosuppressive function. These results provide a clearer picture of how to enhance MSC immunomodulation for clinical use.

## Introduction

Mesenchymal stem cells (MSCs) are multipotent progenitor cells which have the potential to differentiate into osteocytes, adipocytes, and chondrocytes [1]. In addition to their regenerative properties, MSCs also have significant immunosuppressive potential [2–4]. MSCs are known to have a role in dampening the innate immune response, by inhibiting maturation and antigen-presenting capacity of dendritic cells [5–7], and decreasing proliferation and cytotoxicity of natural killer cells [8,9]. MSCs also suppress the adaptive immune response, by dampening both CD4+ helper and CD8+ cytotoxic T cell proliferation and exertion of their respective functions [10–13].

While these pathways have been relatively well delineated, the effect of MSCs on regulatory T cell (Treg) populations remains less well described. A handful of groups have described an increase in Treg expansion in response to MSC exposure [14–16]. However, the exact mechanism by which MSCs exert this effect on Tregs is yet unknown. Also unknown are the microenvironmental conditions that influence this interaction between MSCs and Tregs.

Fully defining the role of MSCs and their interaction with Tregs is of importance in the use of MSCs in prevention of acute rejection in transplantation. The immunosuppressive potential of MSCs has been demonstrated in several animal models, including skin grafts, solid-organ transplants, graft-versus-host disease, and most recently vascularized composite allotransplantation [17–27]. However, the widespread use of MSCs in transplant tolerance comes with several challenges. First, although few mediators and mechanisms have been proposed [7,12,26], the complete mechanism by which MSCs exert their immunosuppressive function remains unclear. Secondly, MSCs are not innately immunosuppressive, and must be stimulated or primed to exert these immunosuppressive effects [3]. An additional challenge lies in the need for continual self-renewing capacity of MSCs without loss of their stem-like properties. Much of the therapeutic potential relies on the ability of MSCs to maintain their stemness over the life of an allograft.

In this study, we look at microenvironmental factors that can prime MSCs for optimal immunosuppressive function while maintaining stem-like characteristics. Because MSCs naturally reside in the bone marrow, which is hypoxic [27–30], priming MSCs in low oxygen tension may increase their immunosuppressive function. While studies have established the effect of hypoxia on increasing MSC proliferation [31–33], the effects on immune properties have yet to be fully established. Additionally, there is evidence that proinflammatory cytokines lead to an increase in MSC-mediated immunosuppression [3,34,35]. Therefore we propose that priming MSCs in low oxygen tension and with an inflammatory microenvironment will increase immunosuppressive potential. Namely, we focus on how these two microenvironmental conditions affect the interaction of MSCs with Tregs.

## Materials and methods

### Animal research

Lewis rats were obtained from Charles River Laboratories (Wilmington, MA) and maintained in the facilities at NYU School of Medicine, in compliance with IACUC policies and guidelines and approval for protocol 160702. Prior to harvesting bone marrow aspirates or spleens, rats were euthanized using carbon dioxide asphyxiation, followed by a bilateral pneumothorax.

### Cell culture

Bone marrow derived-mesenchymal stem cells were obtained from male Lewis rats from femur, tibia, humerus and pelvis, as described [36]. Cells were cultured in Mesencult (Stemcell technologies, Vancouver, BC) in either normoxia (21% oxygen), hypoxia (5% oxygen) or near anoxia (0.5% oxygen). MSCs were plated at 3,000 cells/cm^2^, passaged at 80% confluency, and analyzed at passages 3 through 5. For mechanistic studies, MSCs were treated with 1-methyl-D-tryptophan (1-MT), a competitive inhibitor of indolamine 2,3-dioxygenase (IDO).

### Cell proliferation assays

Doubling time was measured by counting trypsinized cells using a hemocytometer. Cells were trypsinized after reaching 80% confluency, at passages 1–5. Proliferation was determined using an MTT Cell Proliferation Assay Kit (Invitrogen, Waltham, MA). Briefly, cells were seeded in a 96-well plate, cultured for 10 days, incubated with MTT for 4 hours, and measured spectrophotometrically.

### Migration assays

Migration was studied by modified transwell assays. MSCs were plated onto transwell inserts, with no cytokines or IFNγ and/or TNFα. SDF-1 was added to Mesencult in the lower chamber. After a 6 hour migration period, non-migrating cells were wiped from the top filter surface. An MTT assay, using the aforementioned kit, was used to determine relative cell numbers, as a measure of migration.

### Cell differentiation

MSCs at passages 3–5 were grown in either hypoxia or normoxia with adipogenic differentiation media of Mesencult + 0.25mM IBMX (Sigma-Aldrich, St. Louis, MO) + 1μM dexamethasone (Sigma-Aldrich, St. Louis, MO) + 1mM insulin (Sigma-Aldrich, St. Louis, MO) for 4 days, then only Mesencult + 1mM insulin for 10 days. Similar MSCs were also grown in osteogenic differentiation media of Mesencult + 10mM β-glycerophosphate (Sigma-Aldrich, St. Louis, MO) + 10nM dexamethasone + 100μM ascorbic acid (Sigma-Aldrich, St. Louis, MO) + 2mM L-glutamine (Fisher Scientific, Pittsburgh, PA) for 14 days. For chondrogenic differentiation, 200,000 cells were pelleted at 300g for 10 min, and grown in chondrogenic differentiation media of Mesencult basal media + 1% FBS + 100nM dexamethasone + 50μg/ml ascorbic acid + 40μg/ml L-proline (Sigma-Aldrich, St. Louis, MO) + 1% ITS supplement (Sigma-Aldrich, St. Louis, MO) + 1mM sodium pyruvate (Fisher Scientific, Pittsburgh, PA) + 10ng/ml TGFβ-3 (Shenandoah Biotechnologies, Warwick, PA) + 100ng/ml BMP-2 (Shenandoah Biotechnologies, Warwick, PA) for 14 days.

### Immunohistochemistry

Normoxic or hypoxic MSCs were stained for morphology with H&E at passage 3. Staining for adipogenic differentiation using Oil Red O and for osteogenic differentiation using Alizarin Red S also performed. Pellets were collected, fixed and processed into paraffin, then slides were stained for osteogenic differentiation with fast green and Safranin O. Images were collected using a Nikon DS-Fi1 camera (Nikon Corporation, Tokyo, Japan) on an Olympus BX51 microscope (Olympus, Tokyo, Japan) using NIS Elements software (Nikon Corporation, Tokyo, Japan) and processed using Adobe Photoshop CC.

### RNA extraction and RT-PCR analysis

RNA was isolated from cell pellets using an RNeasy Mini Kit (Qiagen, Venlo, Netherlands). cDNA was generated using a high capacity cDNA synthesis kit, from 500ng mRNA (Applied Biosystems, Grand Island, NY). Real-time PCR was performed using SYBR Green Master mix (Life Technologies, Grand Island, NY). Primers were designed, and obtained from Life Technologies.

### Phenotyping

MSCs were stained for 30 minutes at 4°C with anti-CD90-FITC, anti-CD29-PE, anti-CD11b-PerCP, anti-CD45-APC (eBioscience, San Diego, CA). Cells were washed and resuspended with 1xPBS +2% BSA + 0.1% NaN_3_, and analyzed for presence of these cell surface markers using flow cytometer. 10,000 events were collected using a FACS Calibur cytometer (BD Biosciences, San Jose, CA), and data was analyzed using FlowJo software (FlowJo LLC, Ashland, OR).

### Kynurenine assay

A kynurenine assay was performed using supernatants from normoxic or hypoxic MSCs. 50μl of supernatant was mixed with 50μl of 30% (w/v) trichloroacetic acid, incubated for 30 minutes at 50°C, centrifuged at 10,000g for 10 minutes and mixed in a 1:1 ratio with freshly prepared Ehrlich’s reagent (4% p-dimethylaminobenzaldehyde in glacial acetic acid) in a 96-well clear flat bottom plate. Kynurenine standard serial dilutions were also prepared and mixed with Ehrlich’s reagent in a 1:1 ratio. The plate remained at room temperature for 10 minutes, and was read on a plate reader at 490nm.

### MSC/T cell/endothelial cell (EC) co-cultures

Spleens were excised from Lewis rats, and CD4+ T cells were isolated using magnetic cell sorting (MACS separator, Militenyi, San Diego, CA), according to manufacturer’s protocol. Briefly, splenocytes were resuspended in 1xPBS + 1%BSA, and labeled with 10ul anti-rat CD4 microbeads/10^7^ cells. CD4+ cells were then magnetically sorted with MACS LS separator columns. CD4+ cells were co-cultured with passage 3 normoxic or hypoxic MSCs, and passage 2–3 allogeneic rat aortic ECs (VEC Technologies, Rensselaer, NY) in 24-well flat-bottom plates. CD4+ cells were plated 24 hours after MSCs and ECs in a MSC:EC:CD4+ cell ratio of 1:20:20. CD4+ cells were cultured with MSCs, ECs, or both. Additionally, each combination of cells was plated with transwell inserts to separate the different cell types. Cultures were maintained in a 1:1 ratio of Mesencult and Endothelial Cell Growth Medium (Lonza, Basel, Switzerland) with rhTGFβ (20ng/ml), rhIL-2 (300U/ml) (Shenandoah Biotechnologies, Warwick, PA), and anti-CD3 (2.5μg/ml) (Fisher Scientific, Pittsburgh, PA) for 48 hours in hypoxic conditions. Cells were stained with anti-CD4 and anti-FoxP3 antibodies (Biolegend, San Diego, CA) using a FoxP3 Staining Buffer Kit (eBioscience, San Diego, CA). Flow cytometry for CD4 and FoxP3 was performed and analyzed as above, for CD4+ cells that stained FoxP3+ using a ZE5 Cell Analyzer (Propel Labs, Fort Collins, CO).

### Cytospin and Immunocytochemistry

CD4+ splenocytes from co-cultures were collected in chilled 1.5mL Eppendorf tubes and counted. Following a brief centrifugation at 300*xg* and 4°C, cells were resuspended to 10^5^ cells/200μL in cold 2%FBS/PBS. Using a Cytospin 4 (ThermoFisher Shandon, CA), 200μL of cell suspension was loaded and centrifuged at 1000rpm (113*xg*) for 3 minutes. Slides were immediately fixed in 4% paraformaldehyde, followed by permeabilization with 0.1% Triton-X-100. Cells were stained for CD4 and FoxP3, and photographed using a Zeiss Observer microsope (Germany).

### Statistical analysis

All data were analyzed using Graphpad Prism (La Jolla, CA). All experiments were performed with at least n = 6, and analyzed using Students’ t-tests and ANOVA for multiple pairwise comparisons. A p-value of < 0.05 determined statistical significance.

## Results

### Hypoxia favors maintenance of MSC stemness features, and maintains differentiation potential

**Fig 1:**
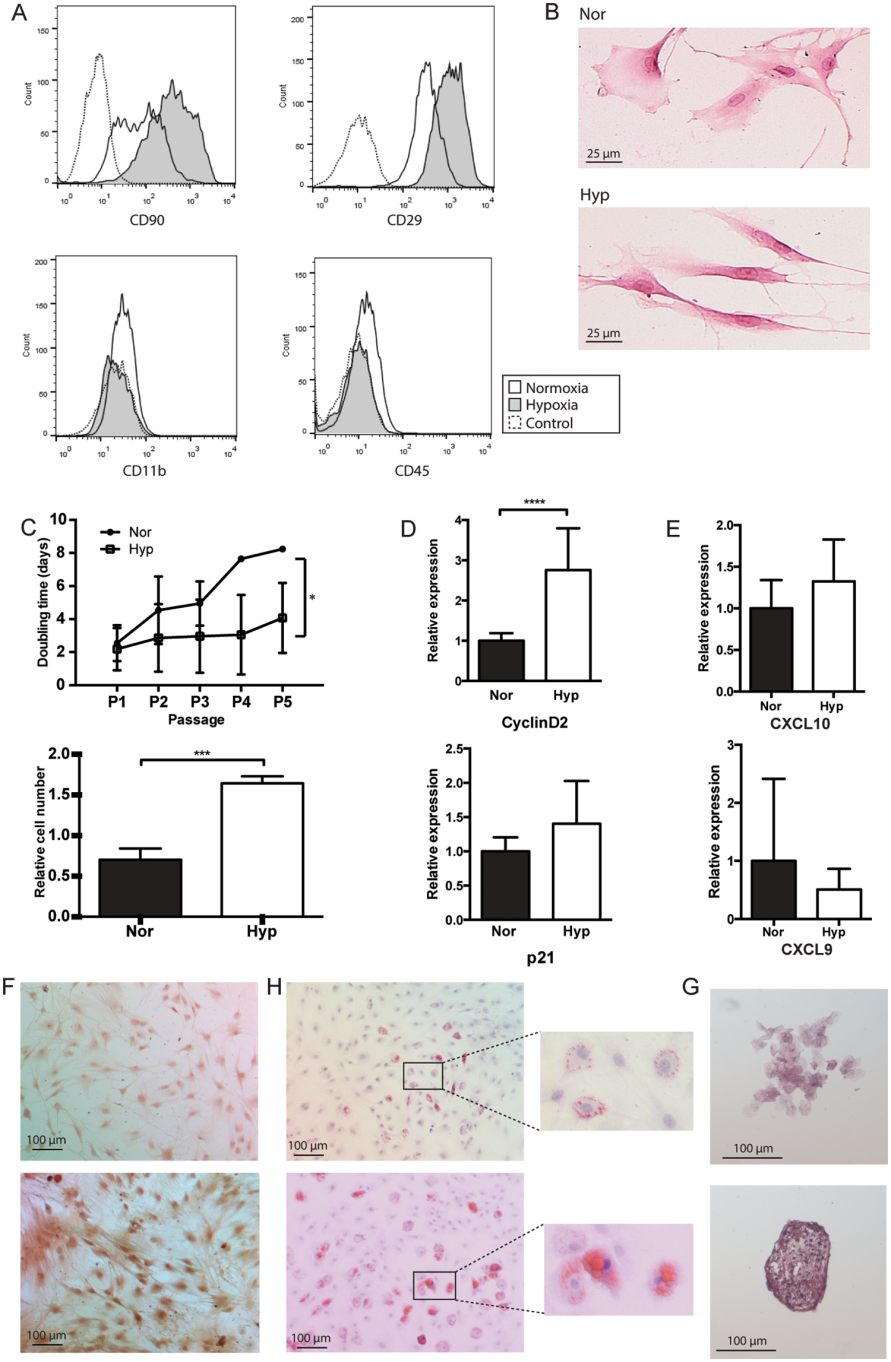
Hypoxic MSCs maintain stemness properties. (A) Flow cytometry of MSCs at passage 3, assessed for positive staining for CD29 and CD90 and negative staining for CD11b and CD45. Normoxic or hypoxic MSCs or unstained control indicated in legend. (B) H&E stain of normoxic (Nor) or hypoxic (Hyp) MSCs. (C) Doubling time of Nor or Hyp MSCs from passage 1–5 (top panel) and relative cell number as determined by MTT assay, over 10 days (bottom panel). (D) Relative expression of cyclinD2 and p21 in Nor or Hyp MSCs at passage 3, n = 6 (E) Nor or Hyp MSCs cultured in osteogenic differentiation media, stained with Alizarin Red S. Passage 4, 10x. (F) Nor or Hyp MSCs cultured in adipogenic media, stained with oil red O. Passage 4, 10x. Inset shows close-up of MSCs. (G) Nor or Hyp MSC pellets cultured in chondrogenic media, stained with safranin O. Passage 4, 10x. (G) Relative expression of CXCL9 and CXCL10 in Nor or Hyp MSCs, n = 6. Results are all expressed as mean ± SD. *p<0.05, ***p<0.001, ****p<0.0001.

We first sought to ensure that growing MSCs under hypoxia conditions maintains stemness, by positive staining for CD90 and CD29, and negative staining for CD45 and CD11b, which are known positive and negative markers of MSCs [37]. Other potential MSC cell surface markers could not be used as those antibodies are currently unavailable in the rat model. We saw a greater increase in both CD90 and CD29 in MSCs grown in hypoxia, as compared to normoxia, while the negative markers CD11b and CD45 remained unchanged (Fig 1A). At passage 3, normoxic MSCs demonstrated 64.9% CD90+/CD29+/CD45-/CD11b- MSCs, and in hypoxia demonstrated 75.8% CD90+/CD29+/CD45-/CD11b- MSCs. At later passages, we found that stemness markers decreased in both normoxia and hypoxia (49.3% and 52.0% CD90+/CD29+/CD45-/CD11b- MSCs at passage 5, respectively), but remained higher in hypoxia. Histologically, hypoxic MSCs maintain their thin spindle “stem-like” shape. Normoxic MSCs demonstrate loss of this morphology, and appear wider and flatter (Fig 1B). This data suggests that culture in hypoxia, even over several passages, promotes maintenance of MSC stem-cell markers.

In investigating the effect of hypoxic culture on MSC proliferation, we found that MSCs cultured in hypoxia, vs normoxia, demonstrate an over 2-fold increase in relative proliferation (normoxia 0.70±0.14 vs hypoxia 1.64±0.09, p<0.001) (Fig 1C). Hypoxic MSCs also demonstrate shorter doubling time over several passages (passage 4: hypoxia 4.07±2.12 days vs normoxia 7.75±0.14 days, p<0.05) (Fig 1C). Furthermore, in hypoxia we found greater expression of cyclinD2 (2.7±1.0 times, p<0.0001), and p21 (1.4±0.6 times), both of which are markers of cell cycle progression (Fig 1D).

Finally, we looked at expression of chemokines known to facilitate MSC immunosuppression, CXCL10 and CXCL9 [3]. Interestingly, we found no difference in expression of these markers between normoxia and hypoxia (Fig 1E).

We then studied whether hypoxic culture affected differentiation potential by growing MSCs in either adipogenic, osteogenic, or chondrogenic differentiation media. We found that MSCs grown under hypoxic conditions in osteogenic differentiation media showed greater staining of calcium deposits with alizarin red (Fig 1F). Hypoxic MSCs grown in adipogenic media stained for greater lipid content with oil red O. We found both a greater number of cells staining with oil red O, as well as larger lipid droplets themselves (Fig 1G). Pellets of MSCs were stained for cartilage content with safranin O, and hypoxic pellets showed greater staining. We also saw a greater deposition of ECM material in the pellet itself, again demonstrating greater chondrogenic differentiation (Fig 1H). This indicates that growth in hypoxia maintains tri-lineage differentiation potential of MSCs, a characteristic of stem-like MSCs.

These data suggest that growth in hypoxia maintains stem-like properties, including the potential for tri-lineage differentiation, and increases proliferation, all properties of MSCs that are involved in exertion of their immunomodulatory functions.

### Near anoxia decreases immunosuppressive potential

**Fig 2:**
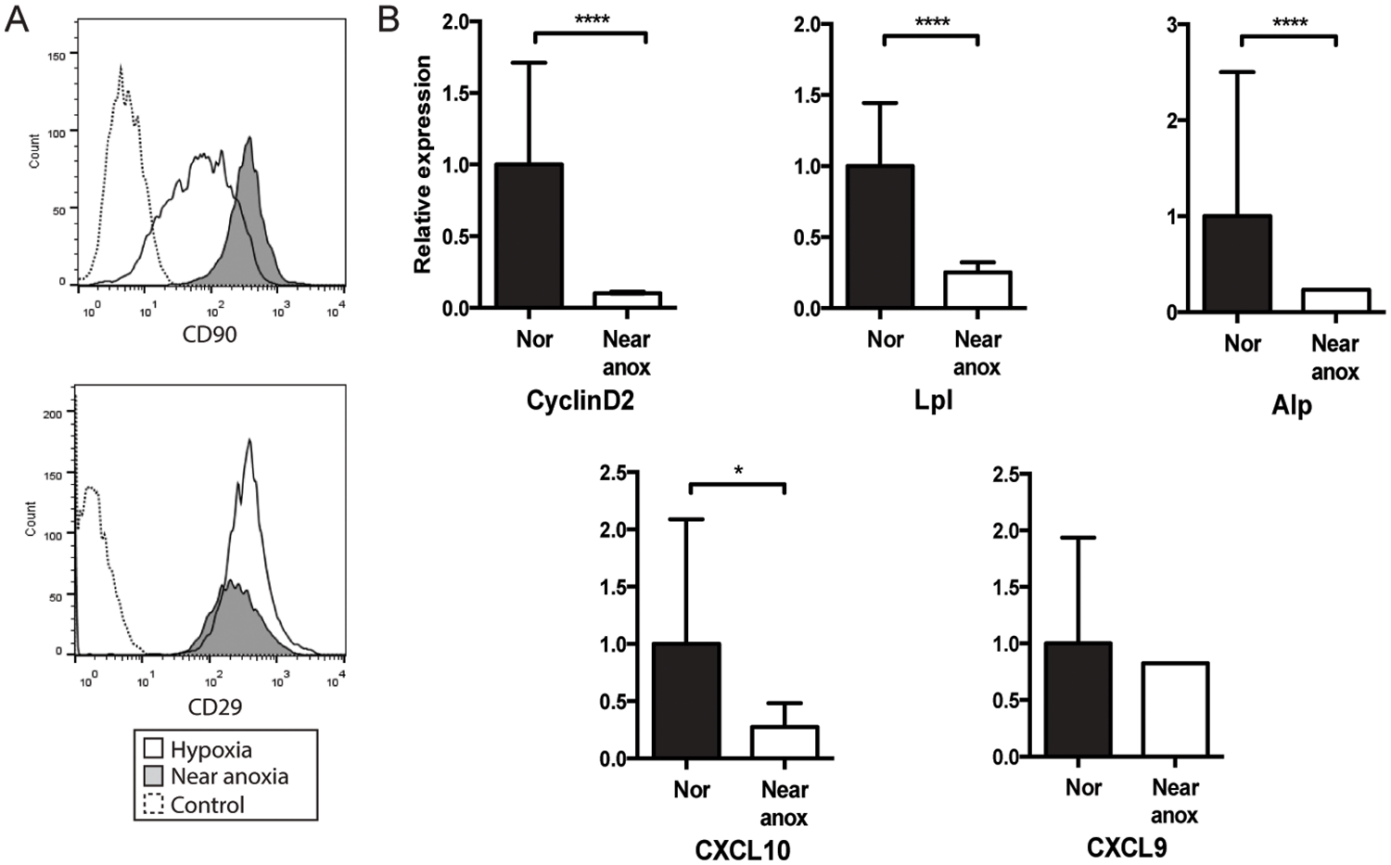
Near anoxia decreases expression of stemness-related markers. (A) Flow cytometry of MSCs at passage 3, assessed for positive staining for CD29 and CD90. Hypoxic or near anoxic MSCs or unstained control indicated in legend. (B) Relative expression of cyclinD2, lpl, alp, CXCL10, CXCL9 in MSCs grown in normoxia (21% oxygen, Nor) or near anoxia (0.5% oxygen, near anox). Results are expressed as mean ± SD. *p<0.05, ****p<0.0001.

Given the profound impact of a low oxygen tension of 5% O_2_ on MSC stemness functions, we next investigated the effect of growth in a near anoxic environment of 0.5% O_2_. When MSCs were cultured in near anoxia, we found an increase in the percent of MSCs expressing CD90, although expression of CD29 remained relatively unchanged. Overall, there was a significant increase in the percentage of CD90+/CD29+/CD45-/CD11b- MSCs (97.9%), indicating that stemness markers are further preserved at very low oxygen tensions (Fig 2A). However, compared to expression in normoxia, markers of proliferation (cyclinD2 10±1% of normoxia, p<0.0001), differentiation (alkaline phosphatase (alp) 25±1%, and lipoprotein lipase (lpl) 23±0%, p<0.0001 for both), and chemokines that facilitate immunosuppressive function (CXCL10 27±21%, p = 0.01 and CXCL9 83%, NS) were significantly decreased (Fig 2B). This data indicates that although near anoxia maintains MSCs stemness markers, it has a negative effect expression of other markers associated with proliferation and immunomodulation.

### Priming in hypoxia enhances MSC stimulation of Treg proliferation

**Fig 3:**
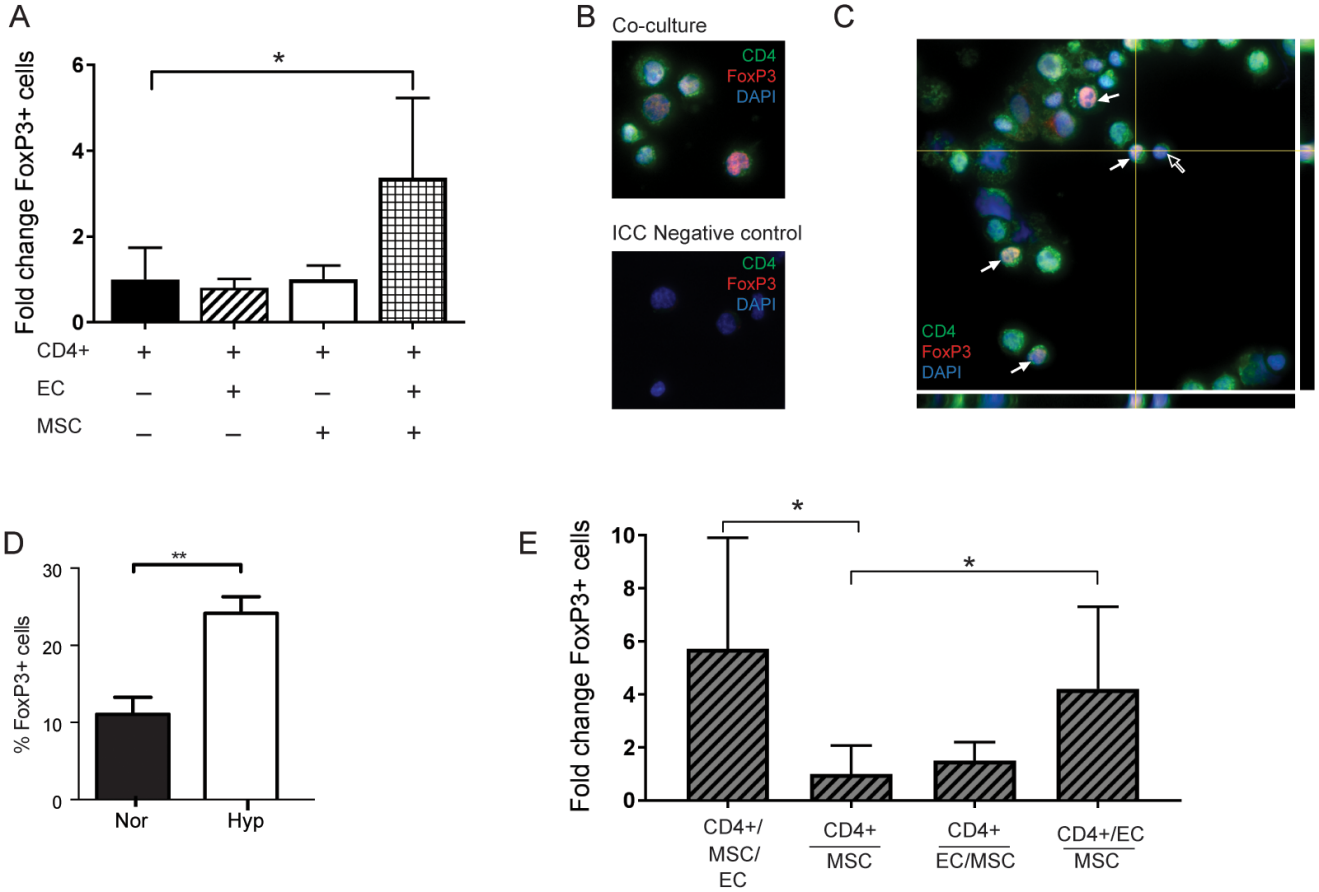
Hypoxic MSCs stimulate regulatory T cell proliferation via paracrine mechanisms. (A) Fold change of FoxP3+ CD4+ cells, in co-cultures with MSCs and ECs (B) Cytospun CD4+ cells from co-cultures, immunostained as indicated. (C) Orthogonal view of immunostained FoxP3+ Tregs. Filled and unfilled arrows, CD4+/FoxP3+ and CD4+ cells, respectively. (D) CD4+ and ECs were co-cultured with either Nor or Hyp MSCs. (E) MSCs, ECs, and CD4+ cells were either co-cultured together or physically separated by a transwell insert. n = 3 for each. Results are expressed as Treg-fold change as compared to CD4+ cells alone. Data is represented as mean ± SD. *, p<0.05, **, p<0.01.

After characterizing MSCs and ensuring that stemness is maintained in our hypoxic MSC cultures, we investigated the mechanisms of MSC immunomodulation. Specifically, we examined the effect of MSCs on Treg proliferation. Using a co-culture systemwith allogeneic ECs, which act as weak antigen presenting cells [38], we found that when CD4+ cells are co-cultured with both MSCs and ECs, they demonstrate a 3.4± 1.9-fold increase in FoxP3+ Tregs than when cultured alone (p<0.05) (Fig 3A). CD4+ cells co-cultured with ECs, or MSCs alone, showed 0.80-fold and 1.02-fold changes in FoxP3+ Tregs (NS), respectively, as compared to CD4+ cells cultured alone. To further verify the expression of FoxP3 in co-cultured CD4+ cells, we analyzed cytospun cells for CD4 and FoxP3 immunoreactivity (Fig 3B), and confirmed CD4+ cells with nuclear FoxP3 (Fig 3C).

We then looked at whether Treg proliferation and differentiation is affected when MSCs are primed in hypoxia. Percentage of Tregs more than doubled when co-culture included hypoxic-primed MSCs, vs normoxic MSCs (24.13±2.16% vs 11.06±2.19% FoxP3+ cells, p<0.01) (Fig 3D).

To determine whether this interaction among hypoxic MSCs, ECs and CD4+ cells requires direct contact, we used transwell inserts in our co-cultures to physically separate the different cell populations. First, we cultured CD4+ cells in an insert overlaying only MSCs, and found significant reduction in FoxP3+ cells, in contrast to the original co-culture conditions (Fig 3E). Then, we cultured CD4+ cells overlaying both MSCs and ECs, and found similar levels of FoxP3+ cells as the previous condition. Interestingly, when ECs were cultured with CD4+ cells in the insert, overlaying MSCs, FoxP3+ cells increased 4.2-fold compared to CD4+ cells in the insert alone. This was comparable to the 5.7-fold change when all three cell types were cultured together with no insert (Fig 3E). Our findings suggest that when co-stimulated by MSCs via a paracrine mechanism, CD4+ cells require direct interaction with ECs to proliferate and differentiate into Tregs.

### IDO expression increases in hypoxia, and may mediate MSC immunomodulation

**Fig 4:**
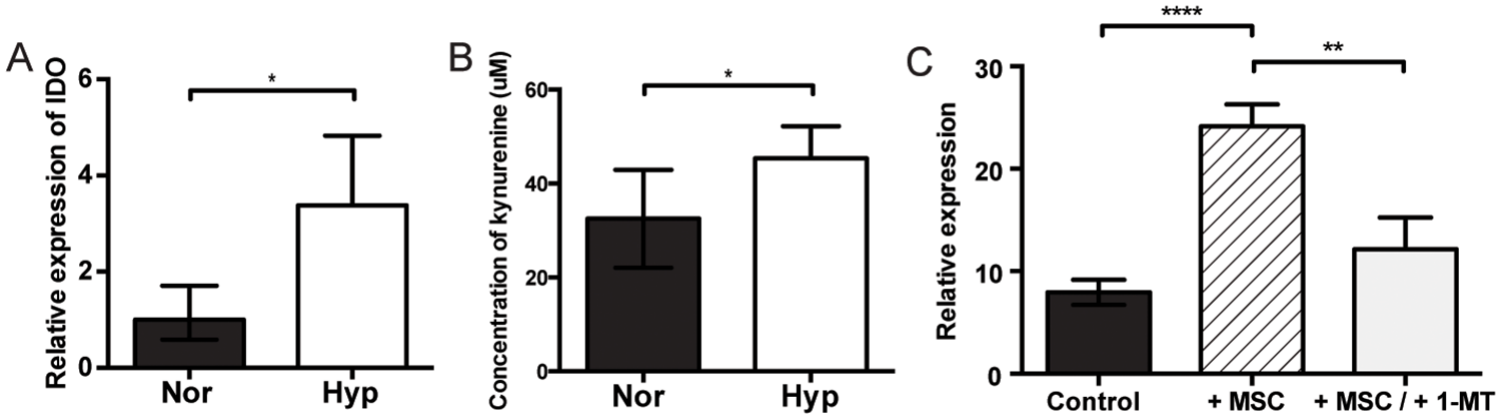
IDO expression increases in hypoxia, and may mediate MSC immunomodulation. (A) Relative expression of IDO by either Nor or Hyp MSCs (B) Relative concentration of kynurenine in supernatant of either Nor or Hyp MSCs. (C) CD4+ and ECs were co-cultured either alone (control) or with Hyp MSCs, or with Hyp MSCs exposed to the IDO inhibitor 1-MT. n = 6 for each. Results are expressed as mean ± SD. * p<0.05, ** p<0.01, **** p<0.0001.

After determining that hypoxic enhances MSC immunomodulation via Treg stimulation, we then investigated the role of IDO in this interaction. Because IDO has been shown to mediate aspects of MSC immunosuppression [12, 37], we hypothesized that it may be the mediator by which MSCs, especially those primed in hypoxia, stimulate Tregs. We first wanted to determine whether hypoxic MSCs express more IDO than normoxic MSCs. Hypoxic MSCs demonstrated significantly greater IDO expression (3.38±1.45 times that of normoxia, p<0.05) (Fig 4A). Additionally, hypoxic MSCs produced significantly more kynurenine, a metabolite of IDO (hypoxia: 45.36±6.89μM vs normoxia: 32.51±10.41μM, p<0.05) (Fig 4B). However, in near anoxia, MSCs demonstrated undetectable expression of IDO.

After confirming that hypoxic MSCs indeed have a greater IDO expression, we wanted to determine whether IDO mediated the effect of MSCs on Treg proliferation. We inhibited the activity of IDO by culturing hypoxic MSCs with the competitive inhibitor 1-MT, and found that proliferation of Tregs significantly decreased (12.14±3.10% vs 24.13±2.16% FoxP3+ cells, p<0.01). In fact, inhibition of IDO almost negated the effects of the addition of MSCs on Treg proliferation (Fig 4C).

### Inflammatory conditions decrease MSC proliferation but increase immunosuppressive potential

**Fig 5:**
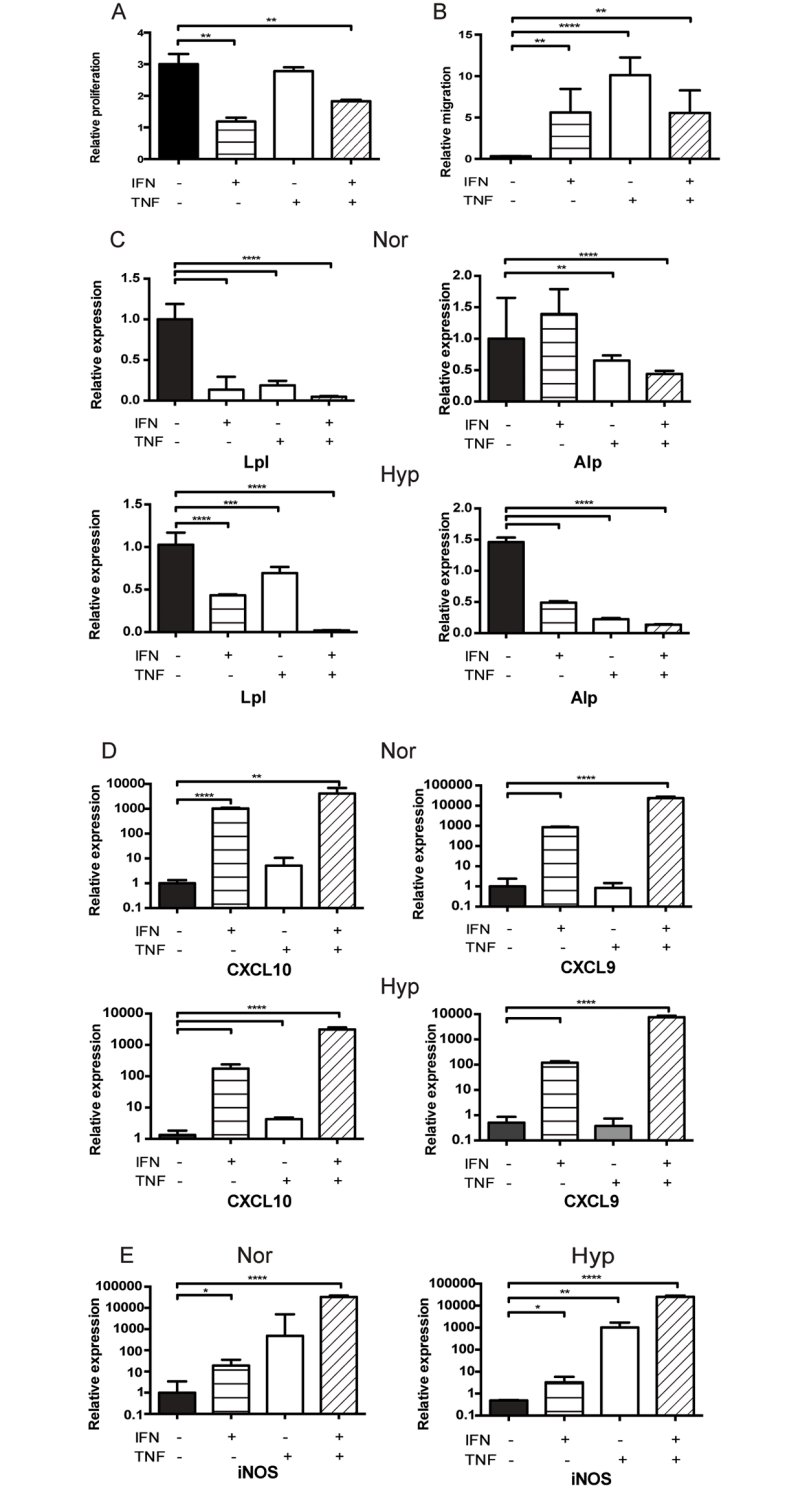
Inflammatory conditions increase stem-like and immunosuppressive markers of MSCs. (A) An MTT assay showing relative proliferation of Nor MSCs, exposed to 24 hours of IFNγ and/or TNFα. (B) Relative migration of Nor MSCS exposed to IFNγ and/or TNFα, assessed by Transwell assay. (C) Relative expression of lpl or alp in Nor (left two panels) or Hyp (right two panels) MSCs at passage 3. High dose (1000U/ml), 7 day exposure. (D) Relative expression of CXCL10 or CXCL9 in Nor (left two panels) or Hyp (right two panels) at passage 3. High dose (1000U/ml), 7 day exposure. (E) Relative expression of iNOS in Nor (left panel) or Hyp (right panel) at passage 3. High dose (1000U/ml), 7 day exposure. n = 6 for each. Results are expressed as mean ± SD. *p<0.05, **p<0.01, *** p<0.001, **** p<0.0001.

We then investigated the effect of inflammatory conditions on MSC immunomodulation and stem-like characteristics. MSCs exposed to any combination of cytokines demonstrated decreased proliferation, but was significant only when IFNγ was included (vs 100% with no cytokine exposure: IFNγ 39%, p<0.01, IFNγ/TNFα 61%, p<0.01, TNFα 93%, NS) (Fig 5A). However, migration of MSCs increased with exposure to any combination of cytokines: 16-fold with IFNγ (relative migration 5.61±2.84 vs no cytokines 0.34±0.02), 30-fold with TNFα (10.12±2.14), and 16-fold with IFNγ/TNFα (5.57±2.72) (p<0.01 for all). Migration was less when exposure included IFNγ, vs TNFα alone (p<0.05) (Fig 5B).

Normoxic MSCs exposed to almost every combination of cytokines show significant decrease in markers of differentiation in lpl (vs 100% with no cytokine exposure: IFNγ 13±16%, TNFα 19±5%, IFNγ/TNFα 5±1%, p<0.0001 for all) and alp (IFNγ NS, TNFα 65±8%, p<0.01, IFNγ/TNFα 43±5%, p<0.0001). Hypoxic MSCs also show significant decrease with exposure to any combination of cytokines, in lpl (IFNγ 43±1%, p<0.0001, TNFα 69±11%, p<0.001, IFNγ/TNFα 2±1%, p<0.0001) and alp (IFNγ 49±3%, TNFα 22±3%, IFNγ/TNFα 14±1%, p<0.0001 for all) (1000U/ml, 7 day exposure) (Fig 5C). Comparing conditions, there is a greater magnitude of decrease of differentiation markers with IFNγ and TNFα alone in normoxia but a greater magnitude of decrease in hypoxia with IFNγ/TNFα.

Exposure of normoxic MSCs to IFNγ or IFNγ/TNFα results in significant increase in CXCL10 (IFNγ 1037±69-fold, p<0.0001, IFNγ/TNFα 4116±2659-fold, p<0.01) and CXCL9 (IFNγ 855±63-fold, p<0.0001, IFNγ/TNFα 23343.50±4098.00 times, p<0.0001). There was no increase when exposed to TNFα alone. A similar increase was seen in hypoxic MSCs (CXCL10: IFNγ 173±59-fold, TNFα 4.3 ±0.5-fold, IFNγ/TNFα 3099±477-fold, p<0.0001 for all. CXCL9: IFNγ 121±16-fold, p<0.0001, TNFα NS, IFNγ/TNFα 7613±1016-fold, p<0.0001) (Fig 5D). The magnitude of increase in expression after cytokine exposure was generally greater in normoxia than in hypoxia.

Additionally, exposure to inflammatory conditions in both normoxia and hypoxia increases the expression of iNOS, a vasoactive molecule. This vasodilation allows for the trafficking of lymphocytes to MSCs by chemokines (Normoxia: IFNγ 18.92±16.04 times, p<0.05, TNFα 478.93±4352.91 times, NS, IFNγ/TNFα 32707.48±5307.37 times, p<0.0001. Hypoxia: IFNγ 3.28±2.39 times, p<0.05, TNFα 1019.87±659.91 times, p<0.01, IFNγ/TNFα 25261.75±3108.44 times, p<0.0001) (1000u/ml, 7 day exposure) (Fig 5E). Overall, our data indicate that despite negative effects on proliferation, microenvironmental inflammatory exposure increases expression of molecules by MSCs that facilitate recruitment of lymphocytes for immunomodulation.

### Inflammatory conditions lead to increased IDO expression, however Treg proliferation remains unchanged

**Fig 6:**
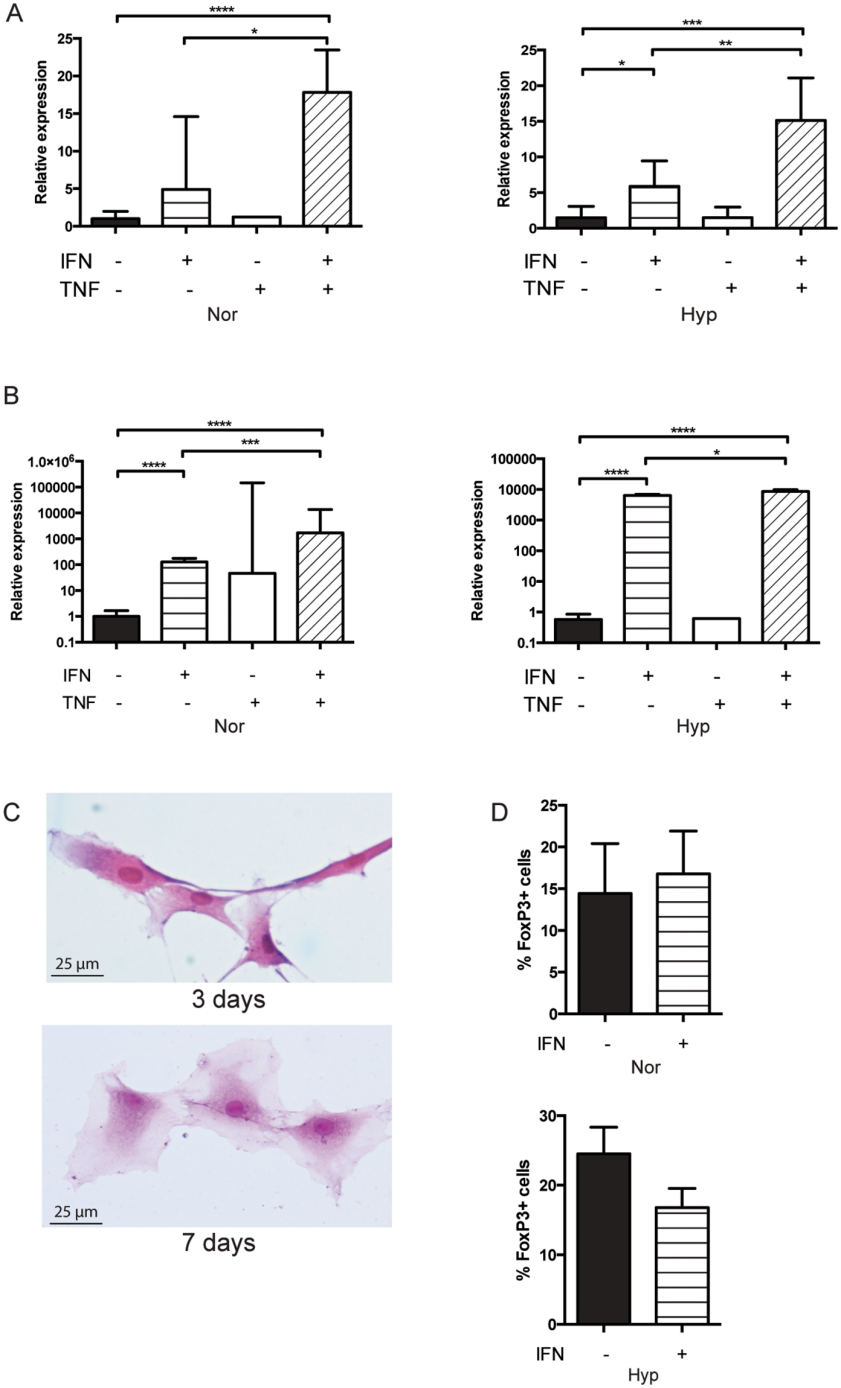
Inflammatory conditions lead to increased IDO expression, however Treg proliferation remains unchanged. (A) Relative expression of IDO in either Nor or Hyp MSCs after 3 days of exposure to IFNγ and/or TNFα (passage 3, 1000U/ml). (B) Relative expression of IDO in either Nor or Hyp MSCs after 7 days of exposure to IFNγ and/or TNFα (passage 3, 1000U/ml). (C) H&E stain of Hyp MSCs with either 3 days or 7 days of IFNγ exposure. 1000U/ml cytokine exposure. (D) CD4+ and ECs were co-cultured with MSCs that were or were not exposed to IFNγ, high dose (1000U/ml), 3 days of exposure. n = 6 for each. Results are expressed as mean ± SD. *p<0.05, ** p<0.01, *** p<0.001, **** p<0.0001.

After determining that inflammatory exposures increase markers of immunomodulation in MSCs, we turned to look at the role of inflammatory conditions in IDO expression and Treg stimulation. When exposed to IFNγ for 3 days, expression of IDO significantly increased. In normoxia, IDO expression increased 17.8±5.6 times when exposed to IFNγ/TNFα (p<0.0001) and increased 4.9±3.3 times with IFNγ only, although this difference was not significant. In hypoxia, IDO expression increased 5.9±3.6 times with IFNγ (p<0.05) and 15.1±5.7 times with IFNγ/TNFα (p<0.001). IFNγ/TNFα increased IDO significantly more than IFNγ alone, in both normoxia (p<0.05) and hypoxia (p<0.01). When MSCs were exposed to TNFα alone, IDO expression was unchanged (Fig 6A).

Longer exposure to either IFNγ or IFNγ/TNFα for 7 days increased IDO expression to a significantly greater level. In normoxia, IDO expression increased 127.2±48.9-fold with IFNγ exposure, and 1711.1±12024.5-fold with IFNγ/TNFα (p<0.0001 for both). In hypoxia, IDO expression increased 6338.1±598.8-fold with IFNγ, and 8601.3±1245.0 –fold with IFNγ/TNFα (p<0.0001 for both). Again no change was seen with TNFα alone (Fig 6B).

However, we did find a negative impact of longer exposure to cytokines on MSC morphology. After 3 days of exposure, MSCs still maintain their spindle-like characteristics. After 7 days, MSCs begin to look wider and flatter (Fig 6C). Our findings indicate that although inflammatory cytokines increase MSC immunomodulatory potential and secretion of IDO, long-term priming must be balanced with potential negative effects on MSC stem-like characteristics.

Given the significant increase in IDO expression by MSCs when exposed to inflammatory conditions, we wondered if this would impact the ability of MSCs to stimulate Treg proliferation. Surprisingly, exposure of MSCs to IFNγ had no impact on the percentage of Tregs, as compared to culture with unexposed MSCs, in both normoxia (16.78±5.12% vs 14.42±5.98% FoxP3+ cells, NS) and hypoxia (16.78±2.75% vs 24.52±3.82% FoxP3+ cells, NS) (Fig 6D).

## Discussion

MSC-mediated immunosuppression provides a powerful potential tool for decreasing the incidence and severity of acute rejection of transplanted allografts [2,4,7,39]. MSC immunosuppressive potential is not innate, but must be induced by environmental factors [3]. A complete picture of these factors is yet to be fully developed. Our study examined a range of environmental factors that may influence MSC-mediated immunosuppression. Here we describe the impact of environmental stimulation on MSC immunosuppression, and further elucidate the mechanism by which MSC immunosuppression is facilitated, by specifically looking at the interactions between MSCs and Tregs and the role IDO plays in this stimulation.

We found that 5% hypoxic conditions, similar to oxygen tension in the native bone marrow environment [27–29], favors MSC-mediated immunosuppression. We first demonstrated that growth in hypoxia increases MSC proliferation. Other groups have also described increased proliferation in hypoxia, although most have studied MSCs at lower oxygen tension [31–33]. We found that extremely low oxygen tensions of 0.5% lead to decreased proliferation. In fact, we find a decrease of expression of all markers studied at very low oxygen tensions of 0.5%, suggesting that MSCs may not be able to exert their immunomodulatory functions well at near anoxic tensions. Overwhelmingly, studies support our finding of increased proliferation in hypoxia, and our data suggest that 5% O_2_ is sufficient to prime MSCs for an immunomodulatory phenotype.

We found that stem-like characteristics are maintained in hypoxia. This is of special importance as any potential therapy using MSCs hinges on MSCs being able to maintain their stem-like state. Our findings that a greater percentage of MSCs express typical stem cell markers in hypoxia, and that hypoxic MSCs phenotypically look more spindle-like, a typical characteristic of MSCs, support this point. Although we considered the possibility that increased proliferation under hypoxic conditions may lead to loss of stem-like properties of MSC, our observations indicate that this is not the case–despite increased proliferation, hypoxic MSCs maintain stemness characteristics better than those grown under normoxic conditions. Our results are supported by other studies showing the maintenance of stemness of despite proliferation is typical of bone marrow-derived MSCs [40,41].

Additionally, at the range of passages studied, hypoxic MSCs retain a greater tri-lineage differentiation potential than normoxic MSCs. Although some studies in the literature question the potential of hypoxic MSCs to differentiate [42,43], others have described that both adipogenic and osteogenic potential still exists [27,31,44,45]. Our results are consistent with maintenance of differentiation potential of hypoxic MSCs, further indicating the maintenance of stem-like characteristics.

To investigate the effect of hypoxia on MSC immunosuppressive potential, we looked at CXCL9 and CXCL10, chemokines that recruit helper and effector T cells to the site of rejection [46,47], thereby facilitating the ability of MSCs to suppress T cells. Interestingly, in 5% oxygen, we saw no change in expression of these chemokines, consistent with a previous report also describing no increase in chemotactic potential [48]. Although hypoxic MSCs do not demonstrate an increase in these chemokines, our co-cultures did demonstrate an increase in Treg expansion when MSCs were primed in hypoxia, suggesting that hypoxic conditions may potentiate the regulatory arm, and not the effector arm, of the T cell immune response.

Much of our study focused on the interactions of MSCs and Tregs. Although the effects of MSCs on cytotoxic and helper T cells have been relatively well established [10–13], the interactions of MSCs with regulatory T cells have been less well established. Several studies have described an increase in Treg proliferation with exposure to MSCs [14–16]. However, our study extends beyond these studies in several important ways. Firstly, none of these studies look specifically at the effect of priming MSCs in hypoxia on Treg proliferation. This is especially important as we have demonstrated that MSC immunomodulation is enhanced through hypoxic priming. Ours is the first and most extensive study looking at how hypoxic conditions specifically affect MSC-mediated Treg proliferation. Additionally, the previously mentioned studies use in vitro co-cultures with only peripheral blood monocytes or isolated T cells with MSCs [7,14,16,49]. However, our study is unique in that our co-culture included allogeneic endothelial cells (ECs), which act as co-stimulators for T cells [38]. This is of particular importance as ECs are likely the first cell type a circulating lymphocyte encounters in an allograft. Our results are also unique in that our MSCs and T cells were obtained from syngeneic rats. All aforementioned studies that performed co-cultures utilized allogeneic MSCs and T cells. We believe our experimental model closely mimics a novel approach in transplant tolerance through the utilization of the recipient’s own MSCs to interact with and dampen the recipient acute rejection response. We find convincing evidence that the addition of autologous MSCs to co-culture increases Treg proliferation, and that this proliferation is increased when MSCs are primed in hypoxia.

The increase in Treg proliferation even when MSCs are physically separated from ECs and T cells demonstrates that a soluble factor mediates MSC immunomodulation. This is consistent with reports of MSC mediated immunosuppression via exosomal functions [15,50,51]. Further investigation into the role of exosomes will allow additional characterization and description of this paracrine immunomodulation by MSCs.

We found that IDO is a key mediator of both hypoxia-stimulated and IFNγ-stimulated immunosuppression. The role of IDO has been debated in the literature. We found only one study that investigated the role of IDO in MSCs under hypoxic conditions, which showed no change in IDO expression in hypoxia [52]. However, this study differs from ours in that the authors used adipose-derived MSCs. In contrast to this single study, we present strong evidence that IDO expression is increased in hypoxia, determined not only with mRNA expression, but also with local concentration of kynurenine, an IDO metabolite. Although mouse-derived MSCs seem to not rely on IDO for mediation of immunosuppression [3,26], the role of IDO in human MSCs has been described [12,34,35,53]. Although studies have described the role of IDO in dampening effector T cell proliferation [12,35], few have investigated into the role of IDO in Treg proliferation. We describe a significant role of IDO in rat MSC immunosuppression, through MSC-mediated Treg expansion. We found that inhibition of IDO significantly decreases the proliferation of Tregs, abrogating the effects of MSCs in co-culture. This implies that IDO has a key mechanistic role in MSC-mediated immunosuppression, which is at least partially carried out via Tregs. Because a mechanistic role of IDO has been described in human MSCs, we believe our results in rat MSCs would be translatable to a human model, again with implications for a therapeutic role in transplant biology.

Migration of MSCs, an important aspect of immunomodulation, was increased following exposure to inflammatory cytokines, which is in agreement with previous studies [34,54]. Although we find that TNFα is the major stimulator of migration, IFNγ seems to be the major influence on MSC-mediated immunosuppression. Exposure to IFNγ, with or without TNFα, consistently led to an increase in expression of CXCL10 and CXCL9 and the vasoactive molecule iNOS, all of which allow for recruitment of lymphocytes toward MSCs. The major role of IFNγ we describe here is supported by several other reports [3,34,35]. Interestingly, we did not find any impact of priming with inflammatory cytokines on Treg proliferation. This indicates that inflammatory conditions may stimulate other MSC immunomodulatory functions aside from Treg stimulation. Further studies into other aspects of MSC immunomodulation can delineate the mechanism of effect of inflammation.

Our extensive in vitro analyses give us greater insight into conditions that would optimally prime MSCs for immunosuppression. There are obvious potential applications of priming MSCs for an immunomodulatory phenotype to clinical use. We have shown the efficacy of these processes in rats, and our model can be easily adapted to human allografts in future studies.

## Supporting information

S1 Raw Data Raw data results used to generate Figs 1–6. (PDF)
