# Supplementary material for: Microenvironmental cues enhance mesenchymal stem cell-mediated immunomodulation and regulatory T-cell expansion
Source: PLoS One. 2018 Mar 7;13(3):e0193178. doi: 10.1371/journal.pone.0193178 (PMC5841747; doi:10.1371/journal.pone.0193178)
Supplement: S1 Raw Data — (PDF) [file pone.0193178.s001.pdf]

Fig 1C	Passage	Condition	Doubling time	Mean	SD
	P1	Normoxia	3.592788104	2.54755934	1.08170537
			2.617151945		
			1.432737957		
		Hypoxia	3.091396236	2.18695913	1.27906722
			1.282522026		
	P2	Normoxia	5.040645735	4.53900651	2.04242507
			5.899079389		
			5.225105743		
			1.636135798		
			1.496526937		
			6.588709808		
			4.337540058		
			3.354774264		
			7.272540897		
		Hypoxia	4.881455356	2.85914087	2.04242507
			6.525531632		
			1.636135798		
			1.356915449		
			0.992802396		
			2.235054603		
			2.386090834		
	P3	Normoxia	6.460175594	4.94318988	1.33542068
			4.424309542		
			3.945084513		
		Hypoxia	1.424640328	2.96555934	2.21470992
			1.424640328		
			1.140139852		
			2.523056904		
			2.637354467		
			4.235234242		
			7.373849237		
	P4	Normoxia	7.646869157	7.74543435	0.13939223
			7.843999534		
		Hypoxia	9.318851159	3.05808003	2.40616194
			1.82155981		
			1.914787178		
			1.29491535		
			3.940471718		
			4.338406614		
			2.576421454		

			4.280768507		
			1.568596974		
			1.568596974		
			1.015504619		
	P5	Normoxia	8.240170868	8.24017087	
		Hypoxia	2.164505788	4.07358329	2.11796476
			2.230214913		
			3.290207782		
			6.341493982		
			6.341493982		
Fig 1D	Gene	Condition	dCt		
	CyclinD2	Normoxia	16.85799885		
			16.70700073		
			16.86299992		
			16.61299992		
			16.38600063		
			16.26199961		
		Hypoxia	14.75400066		
			14.67199993		
			14.77100086		
			15.54200029		
			15.54899979		
			15.61600065		
	p21	Normoxia	14.684		
			14.75		
			15.09		
			14.737		
			14.412		
			14.336		
		Hypoxia	13.549		
			13.703		
			13.863		
			14.67		
			14.633		
			14.663		
Fig 1E	Gene	Condition	dCt		
	CXCL10	Normoxia	24.18700027		
			23.9829998		
			23.4460001		

			23.96000004
			23.92199945
			23.93400049
		Hypoxia	22.95900059
			22.76100016
			22.8029995
			24.26399946
			24.22299957
			23.9819994
	CXCL9	Normoxia	30.16600037
			28.23500061
			29.6069994
			29.24000072
			29.8450017
			29.85600138
		Hypoxia	29.22299862
			28.78600168
			27.94600105
			29.75300169
			28.65800095
			29.73400211

Fig 2B	Gene	Condition	dCt
	CyclinD2	Normoxia	18.557
			18.69
			18.69
			19.192
			19.374
			19.233
		Hypoxia	22.35100031
			22.28499985
			22.33999968
			22.08799934
			22.19699907
			22.19800043
	Lpl	Normoxia	19.767
			19.9
			19.731
			19.31
			19.446
			19.485

	Hypoxia	21.418		
		21.161		
		21.311		
		21.705		
		21.863		
		22.09		
alp	Normoxia	20.482		
		20.398		
		20.523		
		19.788		
		20.061		
		20.009		
	Hypoxia	22.686		
		22.015		
		22.271		
		22.259		
		22.244		
		22.402		
CXCL10	Normoxia	26.869		
		27.034		
		27.072		
		29.307		
		28.704		
		29.358		
	Hypoxia	28.884		
		29.581		
		29.811		
		29.505		
		30.689		
		31.106		
CXCL9	Normoxia	31.387		
		29.767		
		30.868		
		30.7		
	Hypoxia	30.95800161		

Fig 3A	Condition	% FoxP3	Mean	SD
	CD4	6.53	7.94	1.22110606
		8.65		
		8.64		
	CD4/EC	8.1	9.276666667	1.15092716

		9.33		
		10.4		
	CD4/EC/MSC	22.6	24.13333333	2.15715862
		23.2		
		26.6		

Fig 3D	Condition	% FoxP3	Mean	SD
	Normoxia	8.69	11.06333333	2.18792901
		13		
		11.5		
	Hypoxia	22.6	24.13333333	2.15715862
		23.2		
		26.6		

Fig 3E	Condition	% FoxP3	Mean	SD
	MSC/EC/CD4	22.6	22.6	5.65063418
		23.2		
		26.6		
		20.2		
		30		
		13.5		
	CD4/EC	25.5	27.76666667	1.9857828
	MSC	29.2		
		28.6		
	CD4	4.03	4.833333333	0.80500518
	MSC/EC	5.64		
		4.83		

Fig 4A	Condition	dCt
	Normoxia	34.048
		32.427
		33.966
	Hypoxia	33.2750015
		32.2949982

Fig 4B	Condition	Absorbance	Concentration	Mean	SD
	Normoxia	0.085	16.3278	32.50908	10.41058
		0.095	23.4666		
		0.113	36.31644		
		0.115	37.7442		
		0.114	37.03032		

		0.124	44.16912		
	Hypoxia	0.115	37.7442	45.35892	6.88687649
		0.142	57.01896		
		0.118	39.88584		
		0.13	48.4524		
		0.123	43.45524		
		0.126	45.59688		
Fig 4C	Condition	% FoxP3	Mean	SD	
	Control	6.53	7.94	1.22110606	
		8.65			
		8.64			
	MSC	22.6	24.13333333	2.15715862	
		23.2			
		26.6			
	MSC/1-MT	15.6	12.14	3.09883849	
		11.2			
		9.62			
Fig 5A	Condition	Treatment	Relative proliferation		
	Normoxia	None	3.1239		
			2.641275		
			3.247075		
		IFN	0.82236		
			1.42164		
			1.32528		
		TNF	2.863989375		
			2.95152		
			2.526103125		
		IFN/TNF	1.5868125		
			1.832025		
			2.07805		
Fig 5C	Condition	Gene	Treatment	dCt	
	Normoxia	lpl	None	16.9769993	
				16.9769993	
				16.5519991	
				16.8720007	
				17.0110002	
				16.9709992	
			IFN	14.4580007	

		15.553
		15.6699991
		15.1100001
		15.5399995
		15.3319998
	TNF	17.2709999
		17.1189995
		17.3429999
		17.2579989
		17.045001
	IFN/TNF	22.5040007
		23.0559993
		22.5910006
		21.1160002
		21.0160003
		20.5369997
alp	None	19.1760006
		19.2159996
		18.8310003
		19.2219992
		19.2110009
		19.3890004
	IFN	20.2260003
		20.322001
		20.4189997
		20.0680003
		20.6040006
		20.2139997
	TNF	21.4370003
		21.4549999
		21.6319995
		21.5289989
		21.4000006
	IFN/TNF	20.9089994
		20.8770003
		21.3189993
		20.2839994
		19.9439998
		19.6540003

Hypoxia	lpl	None	16.8130007
			16.4000001
			16.6929989
			16.9950004
			17.0249996
			17.2069998
		IFN	18.1530004
			18.0919995
			18.0629992
		TNF	17.1530004
			17.3999996
			17.2860003
			17.3459997
			17.7610006
			17.5809999
		IFN/TNF	22.178
			23.3530006
			22.4939995
			22.7520008
			22.4330001
			22.5630007
Hypoxia	alp	None	18.7160006
			18.4380002
			18.5930004
			18.5999999
			18.6800003
			18.7330008
		IFN	20.2629991
			20.1010003
			20.2410007
		TNF	21.1180005
			21.4780006
			21.3609991
			21.190999
			21.5539999
			21.2820001
		IFN/TNF	22.1630006
			22.0979996
			21.9510002
			22.1340008

				22.0069995
				21.9849997
Fig 5D	Conditions	Gene	Treatment	dCt
	Normoxia	CXCL10	None	24.1870003
				23.9829998
				23.4460001
				23.96
				23.9219995
				23.9340005
			IFN	16.9559999
				16.9809995
				16.9609995
				16.5230002
				17.0110002
				16.7589998
			TNF	24.1590004
				23.7539997
				23.9509997
				23.8000007
				24.0430002
			IFN/TNF	11.5819998
				11.5610003
				11.6029997
				10.3870001
				10.52
				10.1400003
		CXCL9	None	30.1660004
				28.2350006
				29.6069994
				29.2400007
				29.8450017
				29.8560014
			IFN	20.4039998
				20.3410001
				20.2319994
				19.8779998
				20.3319993
				20.1090002
			TNF	27.7420006

			27.1499996
			26.8140006
			25.8959994
		IFN/TNF	12.6589994
			12.6400008
			12.9170008
			11.184001
			11.2450004
			11.0560007
Hypoxia	CXCL10	None	22.9590006
			22.7610002
			22.8029995
			24.2639995
			24.2229996
			23.9819994
		IFN	11.5510001
			11.6100001
			11.5660005
		TNF	20.9920011
			21.4029999
			21.2340002
			20.9180002
			21.4870005
			21.3379998
		IFN/TNF	11.2029996
			11.2180004
			10.9759998
			11.1059999
			10.9879994
			11.2469997
	CXCL9	None	29.2229986
			28.7860017
			27.9460011
			29.7530017
			28.6580009
			29.7340021
		IFN	11.9619994
			11.7140002
			11.8589997

			TNF	26.4119992
				27.5419998
				26.7200003
				27.3179998
				27.7969999
				28.1209989
			IFN/TNF	13.8249993
				13.9589996
				13.6609993
				14.0489998
				13.8059993
				14.0500002

Fig 5E	Condition	Gene	Treatment	dCt
	Normoxia	iNOS	None	29.8820009
				31.4410009
				31.4299989
			IFN	24.9699998
				24.921
				25.0560007
				25.2100005
				25.526999
				25.6719999
			TNF	14.6569996
				14.9430008
				14.8730006
				14.2749991
				14.5199995
			IFN/TNF	12.565999
				12.5230002
				12.5279989
				10.7820005
				10.6979995
				10.4910002
	Hypoxia	iNOS	None	27.9970007
				27.9900002
				28.8560009
				31.8619995
			IFN	11.0820003
				11.2210002

				11.2610002
			TNF	24.6880002
				25.2689991
				24.5250006
				24.3989992
				25.250001
				24.5779996
			IFN/TNF	12.710999
				12.5809994
				12.507
				12.6189995
				12.3999991
				12.4000006

Fig 6A	Gene	Condition	Treatment	dCt
	IDO	Normoxia	None	29.335001
				27.9320021
			IFN	26.1019998
				24.8330011
				26.1440001
				25.5840015
				25.9890003
				29.3970008
			TNF	28.3370008
			IFN/TNF	24.1259999
				24.3559995
				25.2359991
				24.5650005
				24.2939997
				24.2919993
		Hypoxia	None	27.3249998
				28.8149991
			IFN	25.3340006
				25.5580001
				25.6969995
				26.7810001
				27.0130014
				26.0829988
			TNF	26.8200016
				28.6590009

	27.7259994
	29.0690002
IFN/TNF	23.9870005
	25.2390008
	24.9049993
	25.1350002
	24.6549997
	24.3589997

Fig 6B	Condition	Treatment	dCt
	Normoxia	None	29.1549983
			29.66400003
			29.37099981
			30.19699955
			28.45499945
			28.22199869
		IFN	21.73200035
			22.0619998
			22.90499926
			22.79999971
			22.67999935
			21.87199926
			21.66100025
			21.60600042
			22.06800032
			22.52100039
			22.14700031
		TNF	27.55399942
			30.05600023
			27.96900082
			27.25399971
			29.01900053
			29.7464993
		IFN/TNF	21.51700115
			20.65200043
			16.22199917
			16.00399923
			16.22199917
			20.99699974
			21.42699909
			21.59400034

			15.15599966		
			14.57499981		
	Hypoxia	None	29.42499924		
			29.6079998		
			30.41400099		
		IFN	24.55699921		
			24.4119997		
			24.8769989		
			25.06699991		
			25.75200081		
			25.1529994		
			16.526999		
			16.48399973		
			16.72800016		
		TNF	29.89400005		
		IFN/TNF	21.12299967		
			21.62300062		
			21.24300098		
			16.08899927		
			16.2310009		
			15.9279995		
			21.30200052		
			21.31000042		
			21.14999962		
			16.36100006		
			15.90800047		
			16.31799984		
Fig 6D	Condition	Treatment	% FoxP3	Mean	SD
	Normoxia	None	8.69	14.418	5.97876409
			13		
			11.5		
			14.5		
			24.4		
		IFN	23.9	17.36	5.12227163
			19		
			20.6		
			11.8		
			11.5		
			13.9		
